# Healthcare Resources Utilization throughout the Last Year of Life after Acute Myocardial Infarction

**DOI:** 10.3390/jcm12082773

**Published:** 2023-04-08

**Authors:** Ygal Plakht, Harel Gilutz, Jonathan Eli Arbelle, Dan Greenberg, Arthur Shiyovich

**Affiliations:** 1 Department of Nursing, Faculty of Health Sciences, Ben-Gurion University of the Negev, Beer-Sheva 84105, Israel; 2 Emergency Department, Soroka University Medical Center, Beer-Sheva 84101, Israel; 3 Goldman Medical School, Faculty of Health Sciences, Ben-Gurion University of the Negev, Beer-Sheva 84105, Israel; 4 Southern District, Maccabi Healthcare Services, Beer-Sheva 84105, Israel; 5 Department of Health Systems Management, Faculty of Health Sciences, Ben-Gurion University of the Negev, Beer-Sheva 84105, Israel; 6 Division of Cardiovascular Medicine, Department of Medicine, Brigham and Women’s Hospital, Harvard Medical School, Boston, MA 02115, USA

**Keywords:** acute myocardial infarction, healthcare resource utilization, costs, prognosis, follow-up

## Abstract

Healthcare resource utilization (HRU) peaks in the last year-of-life, and accounts for a substantial share of healthcare expenditure. We evaluated changes in HRU and costs throughout the last year-of-life among AMI survivors and investigated whether such changes can predict imminent mortality. This retrospective analysis included patients who survived at least one year following an AMI. Mortality and HRU data during the 10-year follow-up period were collected. Analyses were performed according to follow-up years that were classified into mortality years (one year prior to death) and survival years. Overall, 10,992 patients (44,099 patients-years) were investigated. Throughout the follow-up period, 2,885 (26.3%) patients died. The HRU parameters and total costs were strong independent predictors of mortality during a subsequent year. While a direct association between mortality and hospital services (length of in-hospital stay and emergency department visits) was observed, the association with ambulatory services utilization was reversed. The discriminative ability (c-statistics) of a multivariable model including the HRU parameters for predicting the mortality in the subsequent year, was 0.88. In conclusion, throughout the last year of life, hospital-centered HRU and costs of AMI survivors increase while utilization of ambulatory services decrease. HRUs are strong and independent predictors of an imminent mortality year among these patients.

## 1. Introduction

With significant technological and guideline-directed management advancements throughout the last decades, survival of patients presenting with acute myocardial infarction (AMI) has improved significantly [1,2,3,4,5]. Nevertheless, these patients continue to be at increased risk for mortality and morbidity and hence utilize more healthcare services [5,6,7]. Multiple studies among patients with cardiac diseases (e.g., heart failure [HF] and congenital heart disease) demonstrate that health resource utilization (HRU) peaks in the last year of life, accounts for a substantial share of healthcare expenditure compared to other periods of life, and seems to be increasing over the last decade [8,9,10,11]. Furthermore, it has been previously estimated that one-quarter of all Medicare costs (US) goes to treating patients in their last year of life [12].

The aim of this study was to investigate the changes in HRU and costs throughout the last year of life among AMI survivors and to investigate whether the parameters of HRU can predict mortality during the subsequent year.

## 2. Materials and Methods

### 2.1. Study Population

The present study is a retrospective observational analysis of patients with post-AMI-hospitalization at the Soroka University Medical Center (SUMC) throughout 1 January 2002–31 December 2012. SUMC is a tertiary medical center (1400 beds) in the northern Negev area, southern Israel. Encompassing over 50% of Israel’s land, the Negev is a desert and semidesert expanse located in southern Israel. Its population of around 1,302,000 people reside in a combination of urban and rural locales, with the largest metropolitan area being Beer-Sheva, which is home to 220,000 residents.

AMI was defined according to the International Classification of Diseases, Ninth Revision, Clinical Modification (ICD-9-CM) discharge codes: 410.0*–410.6* for ST-elevation AMI (STEMI) and 410.7*–410.9* for Non-ST-elevation AMI (NSTEMI).

Patients were excluded due to the following criteria: (1) death during the first-year post-AMI and (2) patients who were not insured by Maccabi and Clalit (Southern district). Clalit and Maccabi are the two largest health maintenance organizations (HMOs) out of four HMOs in Israel; they are responsible for the care of more than 80% of the population in the investigated region.

The ethics committees of all the participating institutions approved the study, which was performed in accordance with the Helsinki declaration. Patient consent was waived due to a retrospective data analysis.

### 2.2. Study Design

Patient-level data were collected from the computerized medical records of SUMC, the HMOs, and the Ministry of the Interior Population Registry. The data from the different databases were matched using the unique identification number, which was coded into an anonymous identification number prior to data processing. The baseline data for each patient comprised demographic and clinical characteristics, results of blood tests, echocardiography and coronary angiography, and subsequent cardiovascular procedures (i.e., percutaneous coronary intervention [PCI] and coronary artery bypass graft surgery [CABG]). Adherence with guideline-recommended medical therapy throughout the first year after the hospital discharge was defined based on the rate of issued monthly prescriptions, as previously described [13].

The patients were followed-up for up to ten years post-discharge. Patients’ HRU during the year prior to AMI admission and the follow-up period were collected (last updated in December 2015). These data included number of hospital admissions, total length of in-hospital stay (LOS), number of emergency department (ED) visits, primary care, and outpatient consulting clinic visits, as well as other ambulatory services (e.g., various ambulatory diagnostic or therapeutic procedures). The years were classified from the end of follow-up date or death backwards into mortality years (the year prior to death) and survival years (the years preceding the mortality year for patients who died throughout the follow-up period or all the follow-up years in patients that survived the study follow-up period). HRU and costs were calculated per each follow-up year. In addition, costs of these services were calculated in local currency (New Israeli Shekel [NIS]), based on the tariff published by the Israeli Ministry of Health (2017) [14] and converted to United States Dollar (USD) based on currency exchange rates (1 NIS = 0.29 USD). Patients’ mortality data were last updated in November 2018.

### 2.3. Study Groups

The current study focused on post AMI follow-up years that were divided based on patient survival as following: (1) years that ended in patients’ death (mortality years) and (2) years during which the patient survived (survival years).

### 2.4. Statistical Analysis

Statistical analyses were performed using the IBM SPSS Statistics 26 software. Baseline characteristics of the study groups were presented as mean and standard deviation (SD) for continuous variables and *n* (percent) for categorical data. HRU and their related costs were presented as mean and SD. In addition, due to non-symmetrical distribution of these variables, they were also presented in deciles categories.

Comparisons of the investigated HRU/costs between the years of death and years of survival were performed using Student’s t test, Chi square test, and Chi square test for linear trend in the univariate level.

Multivariable analyses included generalized estimating equations (GEE) models, binary logistic type, for the purpose of controlling for repeated measures for each patient. The dependent variable in these models was a “year” (year of death vs. year of survival). Three level models were built. The first level model (“unadjusted”) included the variable of the HRU and costs. The second level models included all the investigated HRU variables together. The third level model included all the above variables in addition to the patients’ baseline characteristics, number of years after AMI, HRU/cost one year before AMI, and adherence with medical therapy during the first-year post AMI. The results of the models are presented as the Odds Ratios (OR) with Standard errors (SE) and 95% confidence intervals (CI). The ability of the investigated parameters to predict the year of death was assessed using receiver operating characteristic (ROC) curve technique and calculation of c-statistic values.

A further sub-group analysis was performed including only the patients that died during the follow-up. In this sub-group of patients, we assessed the trends in annual HRU/cost (which were presented as continuous variables) by year, becoming close to death. Statistical significance of these trends was estimated in the univariate level using the analysis of variance (ANOVA) test for linear trend. In addition, these trends were estimated using GEE models, linear type. A separate model for each investigated HRU and costs was built and the variable of year of “closing” to the death was the dependent variable in these models. Two level models in the sub-group analysis were built. The first level models included the variable of the HRU/cost alone. The second level model included the potential confounders. The results of the models are presented as the B with SE and 95% CI.

For each test, two-sided *p* < 0.05 (two-tailed) was considered statistical significance.

## 3. Results

### 3.1. Study Population and Groups

Overall, 10,992 patients were included in the analysis with a follow-up of 44,099 patients-years. Throughout the follow-up period 2855 (26.0%) patients died. Thus, based on a per-year classification 2885 years were defined as mortality years. The remaining, 41,244 follow-up years were defined as the survival years. Table 1 presents baseline characteristics and comparison between the study groups (mortality and survival years). The mortality years patients were older at the baseline index hospitalization for AMI, with a higher proportion of females, and some cardiovascular risk factors (e.g., diabetes mellitus, hypertension, and renal failure). The prevalence of other cardiovascular risk factors (e.g., obesity, smoking, and dyslipidemia) was significantly lower compared to patients in the years of survival. Non-cardiovascular co-morbidity was significantly more prevalent in the years of death. Furthermore, the prevalence of a more severe coronary artery disease (e.g., three vessel disease), severe left ventricular (LV) dysfunction, significant valvular disease, and non-invasive therapy were more prevalent among the years of death.

### 3.2. HRU and Costs

Throughout the follow-up period the following HRU were recorded: 180,068 hospitalization days, 14,648 ED visits, 572,249 primary clinic visits, 458,227 ambulatory visits, and 63,742 consulting clinics visits. Total costs were 179,026,740 USD.

During each study year more than 40% of patients were admitted to the hospital at least once and about 20% visited the ED. In addition, half of the patients had more than 10 primary clinic visits, more than 5 ambulatory visits, and at least one consulting clinic visit. The total annual cost of examined healthcare services was more than 1968 USD for 50% of the patients (see Table S1 in the Supplementary Materials for the mean values and deciles of annual HRUs and related costs).

HRU and costs according to mortality vs. survival years are presented in Table 2. It is clearly evident that LOS, ED visits and costs were significantly higher during the years ending in patient death vs. survival years, while utilization of ambulatory services was lower.

### 3.3. Multivariable Analysis

When adjustment for repeated measures was performed, the results of the models showed that increased utilization of in-hospital healthcare services was associated with a higher likelihood for mortality during the subsequent year (Table 3, models a). For example, patients who were hospitalized for one or two days were approximately at two-fold higher risk for mortality during the subsequent year, as compared with years without hospitalizations. Similarly, patients who were hospitalized for up to two weeks, had approximately a seven-fold higher risk and patients who were hospitalized for more than two weeks during a year had a seventeen-fold higher risk for mortality during the subsequent year. Similarly, two or more ED visits were associated with a three-and-a-half-fold increase in mortality during the subsequent year. Increased costs were also significantly associated with higher risk for mortality during the subsequent year. However, primary clinic, consulting clinic, and ambulatory visits were associated with significantly reduced risk for subsequent year mortality.

The results of the multivariable model that included all the investigated HRU parameters are presented in Table 3, model b. These results show that all the investigated HRU parameters are strong independent predicators of mortality during the subsequent year with a “dose-response” like effect. While direct association between hospital services (LOS and ED visits) and mortality was observed, the association with ambulatory services utilization was reversed. Similar results were found when additional potential confounders were included into the models (Table 3, models c and e).

### 3.4. Predictive Ability of the Models

An ability for discrimination between mortality and survival during the subsequent year of the multivariate model based on the URS parameters (Table 3 model c) was high, with c-statistics of 0.876. The discrimination ability (c-statistics) of the model included a total cost (Table 3 model e) was 0.811 (see also Figure S1 in the Supplementary Materials).

### 3.5. Trends in HRU Prior to Mortality Years

**Figure 1:**
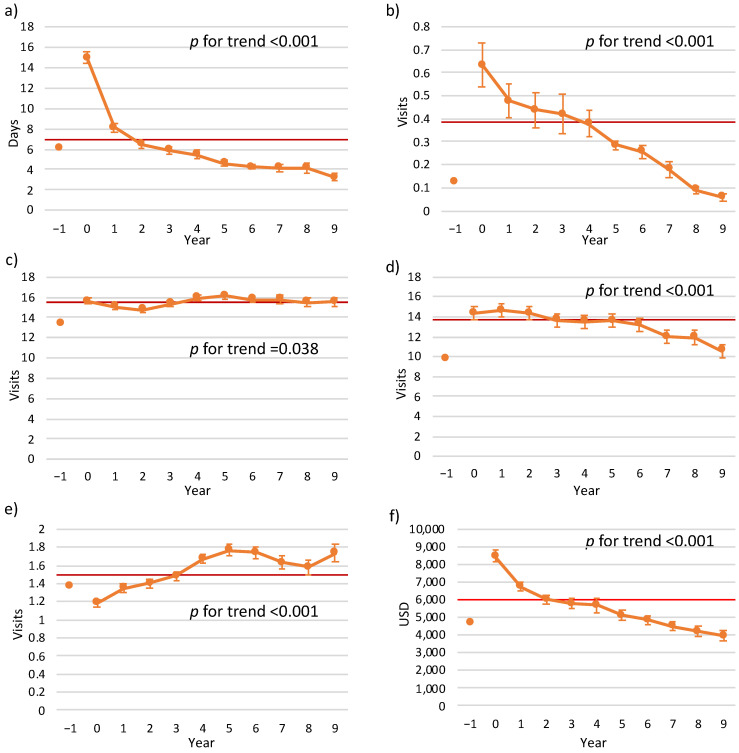
Values of mean (and standard error) of HRUs and costs by year, approaching to a mortality year (this sub-group analysis included only the patients that died during the follow-up period): (**a**) LOS, (**b**) number of ED visits (not resulted hospitalizations), (**c**) primary clinic visits, (**d**) ambulatory visits, (**e**) consultant visits and (**f**) total cost (USD). Category (X axis): (−1)—utilization throughout the year before the index AMI; 0—mortality year; the other categories: years approaching the mortality year. The horizontal line represents the mean utilization throughout the follow-up years, excluding the year preceding the AMI. Abbreviations: AMI—Acute myocardial infarction, ED—Emergency department, HRU—Healthcare resource utilization, LOS—Length of (hospital) stay, USD—United States Dollars.

In order to analyze the trends in HRU towards the year of death, we investigated 2855 patients that died during the follow-up and altogether comprised 13,120 patient-years of follow-up (2885 years of death and 10,265 patient-years prior to mortality). Figure 1 displays the trends in HRU and costs prior to and approaching the mortality year. A trend of increase in annual LOS and ED visits as well as ambulatory visits and a decrease in primary and consulting ambulatory visits were observed towards the mortality year. Furthermore, a trend of increase in the total cost was observed as well.

When adjustment for repeated measurements was performed, the results of the models showed an increase in utilization of in-hospital healthcare services (LOS and ED visits), ambulatory visits and total costs throughout the years approaching the mortality year. No significant trends for the number of primary clinic visits were found. Overall, these findings were consistent when adjusted to potential confounders in multivariable models, except non-significant relationship with the number of ambulatory visits (see Table S2 in the Supplementary Materials).

## 4. Discussion

The current study evaluates changes in HRU and costs throughout the last year of life, as compared with the “years of survival” among AMI survivors, and whether such changes can predict imminent mortality among these patients. The main findings of the current study include: first, throughout the last year of life, greater utilization of hospital healthcare services and costs were observed while utilization of ambulatory services diminished. Second, HRU (both hospital and ambulatory) and their related costs were strong and independent predictors of imminent mortality within a year, with a “dose response” like effect. Third, trends of increase in utilization of hospital services and total cost, beside decline in utilization of ambulatory services throughout the years preceding the last year of life, were observed.

Our findings of significant increase in HRU prior to death, specifically of hospital-based resources, are consistent with previous reports dealing with patients with various morbidities [8,15,16,17]. Although a primary diagnosis of HF or coronary artery disease (CAD), during the last 2-years of life, has previously been reported to be associated with a greater odds of hospitalization over time compared to other primary diagnoses [9], to our knowledge, this is the first report focusing specifically on AMI survivors. Nevertheless, HRU and costs towards the end of life were meticulously investigated among patients with HF, which is often the result of CAD and an AMI [16,17]. Similar to our findings, in patients with HF, HRU and costs were found to increase sharply at the end of life, dominated by hospital care and including specifically hospital admissions (both number of admissions and total LOS) [11]. Furthermore, a trend of increase in HRU was apparent even before the last year of life, similar to our findings [6,11]. Although hospitalization services utilization predominated, ambulatory services were reported to increase as well, contradictory to our findings [6]. This disparity could result from the differences between the studied patients (HF vs. AMI), healthcare systems of different countries, and differences in causes of hospitalization or death. In this context, a recent report by Madelaire et al. [16] found that during the last year of life the majority of in-hospital admissions among HF patients were due to non-cardiovascular causes.

Consistent with our findings, the following characteristics of patients with HF and other chronic illness were associated with greater resource utilization at the end of life: women, elderly (mostly “younger old” age-groups), and patients with greater comorbidity burden [8,9,11,16]. Other reports, however, have shown reduced utilization of hospital-centered healthcare services in the last year of life among very old patients and those with dementia [11,18]. Furthermore, regarding HF, younger patients who die with or of HF receive the costliest care and spend protracted time in-hospitals [17].

Interestingly, previous investigations of temporal trends of healthcare utilization in the last months of life (both HF patients and non-HF patients), showed inconsistent findings: some did not find any changes overtime [19] while others showed a significant decrease [11]. Furthermore, during the last year of life, patients with HF were more often hospitalized for non-cardiovascular than for cardiovascular causes [16].

Although largely unknown, and unanswerable by the design of the current study, several mechanisms can be suggested to explain the observed relationship between HRU throughout the last year of life and imminent mortality among AMI survivors. First, multimorbidity, both cardiovascular, HF in particular, and non-cardiovascular is associated with increased risk for in-hospital HRU prior to death. Second, increased rate of admission might be to treat medical conditions normally treated on an outpatient basis (e.g., respiratory infections and pneumonia, urinary tract infections) due to a perceived increased patient risk due to the previous AMI and associated comorbidity, thus increasing in-hospital and reducing ambulatory healthcare utilization. Third, insufficient ambulatory facilities for end-of-life and palliative care as well as lack of end-of-life discussions with a medical authority and advanced end-of-life directives [20]. Fourth, women with an AMI are more likely to suffer complications and are less likely to receive evidence-based, guideline-recommended medical therapy, to adhere with the recommended therapy and to be followed by a cardiologist, which could explain the long-term and prior to death increased in-hospital healthcare utilization [21,22,23,24,25,26,27,28,29].

### Limitations

Our study has several limitations. First, the retrospective methodology does not enable investigation of causality. Second, patients from only a single center and two out of four HMOs (more than 80% of the patients) were included, hence generalizability may be limited. Third, the causes of mortality were not known and particularly the differentiation between cardiovascular and non-cardiovascular etiologies was not possible. Fourth, rapid evolvement of guideline directed medical therapy from 2002–2012 may affect outcomes to some extent. Fifth, data regarding post-AMI rehabilitation treatment was not collected and therefore might be responsible for some unaccounted bias if differed between the groups. We did not account for differences in healthcare between rural and urban areas, as absence of structured and comprehensive palliative care services (e.g., in rural areas as compared to urban areas) may necessitate unique strategies for palliative and home-based care. Sixth, we were unable to distinguish between type 1 and type 2 AMI patients. Type 2 AMI patients have a reduced likelihood of being discharged on guideline-directed medical therapy and a greater likelihood of experiencing complex comorbidities. Finally, costs of in-hospital services in Israel are based on average estimates rather than specific costs representing actual expenditure for a specific patient, hence expenditure could be underestimated for some patients.

## 5. Conclusions

Throughout the last year of life, the in-hospital healthcare resources utilization and costs of AMI survivors increase while utilization of ambulatory services decreases. Furthermore, HRU and costs are strong and independent predictors of imminent mortality during a subsequent year in these patients with a “dose response” like effect. The findings of the current study could assist decision-makers and healthcare providers in prediction of imminent increased risk for mortality among AMI survivors and thus enables tailoring custom made economically sensitive programs to improve patient management and improve resource allocation.

## Figures and Tables

**Table 1:** The baseline characteristics of the study population (10,992 patients, 44,099 person-years), by study group.

Parameter	Study Group		Total	*p*
	Years of Survival	Years of Death		
*n* (Person-Years)	41,244	2855	44,099	
Demographics				
Age (years), Mean (SD)	63.24 (13.09)	73.98 (11.13)	63.94 (13.23)	<0.001
Sex (males)	29,716 (72.0)	1654 (57.9)	31,370 (71.1)	<0.001
Nationality (minorities)	9580 (23.2)	503 (17.6)	10,083 (22.9)	<0.001
Cardiac diseases				
Supraventricular arrhythmias	5186 (12.6)	718 (25.1)	5904 (13.4)	<0.001
CHF	4833 (11.7)	754 (26.4)	5587 (12.7)	<0.001
CIHD	34,024 (82.5)	1989 (69.7)	36,013 (81.7)	<0.001
Cardiovascular risk factors				
Renal diseases	12,262 (29.7)	1518 (53.2)	13,780 (31.2)	<0.001
Diabetes mellitus	14,382 (34.9)	1370 (48.0)	15,752 (35.7)	<0.001
Dyslipidemia	29,349 (71.2)	1666 (58.4)	31,015 (70.3)	<0.001
Hypertension	21,423 (51.9)	1604 (56.2)	23,027 (52.2)	<0.001
Obesity	9977 (24.2)	546 (19.1)	10,523 (23.9)	<0.001
Smoking	18,152 (44.0)	735 (25.7)	18,887 (42.8)	<0.001
PVD	4220 (10.2)	535 (18.7)	4755 (10.8)	<0.001
Other disorders				
COPD	2249 (5.5)	347 (12.2)	2596 (5.9)	<0.001
Neurological disorders	4925 (11.9)	707 (24.8)	5632 (12.8)	<0.001
Malignancy	1013 (2.5)	144 (5.0)	1157 (2.6)	<0.001
Anemia	20,058 (48.6)	1922 (67.3)	21,980 (49.8)	<0.001
Schizophrenia/Psychosis	544 (1.3)	75 (2.6)	619 (1.4)	<0.001
GI bleeding	743 (1.8)	86 (3.0)	829 (1.9)	<0.001
Alcohol/drug addiction	809 (2.0)	63 (2.2)	872 (2.0)	0.363
HRU one year prior to AMI, Mean (SD)				
LOS (Days)	3.54 (8.49)	6.55 (13.24)	3.74 (8.91)	<0.001
Number of ED visits ^a^	0.097 (0.44)	0.122 (0.641)	0.099 (0.451)	0.038
Number of primary clinic visits	10.63 (10.18)	13.69 (12.02)	10.83 (10.33)	<0.001
Number of ambulatory visits	6.79 (13.89)	10.03 (21.19)	7.00 (14.50)	<0.001
Number of consultant visits	1.06 (1.94)	1.33 (2.10)	1.08 (1.95)	<0.001
Total cost (USD)	3174.46 (6045.33)	4825.91 (9156.25)	3281.37 (6306.42)	<0.001
Characteristics of the hospitalization				
Encounter year, 2002–2006	21,516 (52.2)	1710 (59.9)	23,226 (52.7)	<0.001
2007–2012	19,728 (47.8)	1145 (40.1)	20,873 (47.3)	
Type of AMI, STEMI	22,001 (53.3)	1130 (39.6)	23,131 (52.5)	<0.001
Type of treatment, Noninvasive	7861 (19.1)	1368 (47.9)	9229 (20.9)	<0.001
PCI	27,066 (65.6)	1233 (43.2)	28,299 (64.2)	
CABG	6313 (15.3)	254 (8.9)	6567 (14.9)	
Results of echocardiography ^b^				
Echo performance	35,404 (85.8)	1981 (69.4)	37,385 (84.8)	<0.001
Severe LV dysfunction	2770 (7.8)	322 (16.3)	3092 (8.3)	<0.001
LV hypertrophy	1327 (3.7)	146 (7.4)	1473 (3.9)	<0.001
Mitral regurgitation	1596 (4.5)	197 (9.9)	1793 (4.8)	<0.001
Pulmonary hypertension	1893 (5.3)	271 (13.7)	2164 (5.8)	<0.001
Results of angiography ^c^				
Angiography performance	32,221 (78.1)	1449 (50.8)	33,670 (76.4)	<0.001
Measure of CAD, No or non-significant	1587 (4.9)	92 (6.3)	1679 (5.0)	<0.001
One vessel	8453 (26.2)	249 (17.2)	8702 (25.8)	
Two vessels	9154 (28.4)	342 (23.6)	9496 (28.2)	
Three vessels/LM	13,027 (40.4)	766 (52.9)	13,793 (41.0)	
Characteristics of the follow-up				
Compliance to the medical treatment during the first year ^d^	9825 (23.8)	429 (15.0)	10,254 (23.3)	<0.001
Length of follow-up (years), Mean (SD)	4.59 (2.65)	4.60 (2.45)	4.59 (2.64)	0.908
1–5	5636 (13.7)	180 (6.3)	5816 (13.2)	<0.001
5–10	35,608 (86.3)	2675 (93.7)	38,283 (86.8)	

The data are presented as *n* (%) unless otherwise stated. ^a^ Number of emergency department (ED) visits not resulted hospitalizations. ^b^ Among persons with the results of echocardiography. ^c^ Among persons with the results of angiography. ^d^ Compliance to the medical treatment relates to guideline recommended medical therapy during the first year after discharge. Abbreviations: AMI—Acute myocardial infarction, AV—Atrioventricular, CABG—Coronary artery bypass surgery, CAD—Coronary artery disease, CHF—Congestive heart failure, CIHD –Chronic ischemic heart disease, COPD—Chronic obstructive pulmonary disease, ED—emergency department, GI—Gastro-intestinal, HRU—Healthcare resource utilization, IHD—Ischemic heart disease, LOS—length of (hospital) stay, LM—Left main (artery), LV—Left ventricular, PCI—Percutaneous coronary intervention, PVD—Peripheral vascular disease, SD—standard deviation, STEMI—ST segment elevation myocardial infarction, USD—United States Dollars.

**Table 2:** HRU and costs according to years of death and survival years.

Parameter	Percentiles	Values	Study Group		Total	*p*
			Years of Survival	Years of Death		
*n* (Person-Years)			41,244	2855	44,099	
LOS (Days)						
Mean (SD)			3.15 (12.23)	17.59 (27.269)	4.08 (14.16)	<0.001
	10–50	0	28,611 (69.4)	705 (24.7)	29,316 (66.5)	<0.001
	60	1–2	3486 (8.5)	165 (5.8)	3651 (8.3)	
	70	2–5	3339 (8.1)	287 (10.1)	3626 (8.2)	
	80	5–13	3098 (7.5)	542 (19.0)	3640 (8.3)	
	90	≥14	2710 (6.6)	1156 (40.5)	3866 (8.8)	
Number of ED visits ^a^						
Mean (SD)			0.32 (2.24)	0.57 (3.81)	0.332 (2.37)	<0.001
	0–80	0–1	38,444 (93.2)	2270 (79.5)	40,714 (92.3)	<0.001
	90	≥2	2800 (6.8)	585 (20.5)	3385 (7.7)	
Number of primary clinic visits						
Mean (SD)			12.96 (11.30)	13.26 (13.41)	12.98 (11.45)	0.237
	10–30	0–6	11,883 (28.8)	1041 (36.5)	12,924 (29.3)	<0.001
	40–70	6–17	17,181 (41.7)	939 (32.9)	18,120 (41.1)	
	80–90	≥18	12,180 (29.5)	875 (30.7)	13,055 (29.6)	
Number of ambulatory visits						
Mean (SD)			10.29 (21.24)	11.90 (28.90)	10.39 (21.82)	0.003
	10–40	0–3	17,964 (43.6)	1566 (54.9)	19,530 (44.3)	<0.001
	50	3–5	3708 (9.0)	241 (8.4)	3949 (9.0)	
	60–90	≥6	19,572 (47.5)	1048 (36.7)	20,620 (46.8)	
Number of consultant visits						
Mean (SD)			1.48 (2.25)	0.90 (1.95)	1.45 (2.23)	<0.001
	10–50	0–1	19,599 (47.5)	1847 (64.7)	21,446 (48.6)	<0.001
	60–90	≥2	21,645 (52.5)	1008 (35.3)	22,653 (51.4)	
Total cost (USD)						
Mean (SD)			3757.16 (9194.36)	8428.67 (15,178.26)	4059.60 (9761.90)	<0.001
	10–60	<3491	29,470 (71.5)	1401 (49.1)	30,871 (70.0)	<0.001
	70	3491–4765	4101 (9.9)	308 (10.8)	4409 (10.0)	
	80	4765–7300	3995 (9.7)	415 (14.5)	4410 (10.0)	
	90	≥7300	3678 (8.9)	731 (25.6)	4409 (10.0)	

The data are presented as *n* (%) unless otherwise stated. ^a^ Number of emergency department (ED) visits not resulted hospitalizations. Abbreviations: ED—Emergency department, HRU—Healthcare resource utilization, LOS—Length of (hospital) stay, SD—Standard deviation, USD—United States Dollars.

**Table 3:** Risk for a mortality year according to HRU and costs; multivariable models.

Model ^a^	Parameter	Percentiles	Values	OR	(95% CI)	*p*
a.	LOS (Days)	10–50	0	1 (ref.)		
		60	1–2	1.921	(1.615; 2.284)	<0.001
		70	2–5	3.488	(3.025; 4.022)	<0.001
		80	5–13	7.100	(6.305; 7.996)	<0.001
		90	≥14	17.311	(15.638; 19.164)	<0.001
a.	Number of ED visits ^b^	10–80	0–1	1 (ref.)		
		90	≥2	3.538	(3.204; 3.908)	<0.001
a.	Number of primary clinic visits	10–30	0–6	1 (ref.)		
		40–70	6–17	0.624	(0.570; 0.683)	<0.001
		80–90	≥18	0.820	(0.748; 0.899)	<0.001
a.	Number of ambulatory visits	10–40	0–3	1 (ref.)		
		50	3–5	0.746	(0.648; 0.858)	<0.001
		60–90	≥6	0.614	(0.568; 0.665)	<0.001
a.	Number of consultant visits	10–50	0–1	1 (ref.)		
		60–90	≥2	0.494	(0.457; 0.534)	<0.001
b.	LOS (Days)	10–50	0	1 (ref.)		
		60	1–2	2.857	(2.386; 3.421)	<0.001
		70	2–5	5.138	(4.418; 5.975)	<0.001
		80	5–13	10.414	(9.124; 11.885)	<0.001
		90	≥14	25.160	(22.279; 28.413)	<0.001
	Number of ED visits ^b^	10–80	0–1	1 (ref.)		
		90	≥2	2.526	(2.242; 2.844)	<0.001
	Number of primary clinic visits	10–30	0–6	1 (ref.)		
		40–70	6–17	0.527	(0.474; 0.587)	<0.001
		80–90	≥18	0.385	(0.339; 0.437)	<0.001
	Number of ambulatory visits	10–40	0–3	1 (ref.)		
		50	3–5	0.682	(0.586; 0.795)	<0.001
		60–90	≥6	0.585	(0.532; 0.643)	<0.001
	Number of consultant visits	10–50	0–1	1 (ref.)		
		60–90	≥2	0.591	(0.537; 0.650)	<0.001
c.	LOS (Days)	10–50	0	1 (ref.)		
		60	1–2	2.445	(2.032; 2.942)	<0.001
		70	2–5	3.537	(3.029; 4.130)	<0.001
		80	5–13	6.619	(5.768; 7.595)	<0.001
		90	≥14	16.257	(14.281; 18.507)	<0.001
	Number of ED visits ^b^	10–80	0–1	1 (ref.)		
		90	≥2	2.675	(2.346; 3.051)	<0.001
	Number of primary clinic visits	10–30	0–6	1 (ref.)		<0.001
		40–70	6–17	0.565	(0.505; 0.632)	<0.001
		80–90	≥18	0.411	(0.357; 0.474)	<0.001
	Number of ambulatory visits	10–40	0–3	1 (ref.)		
		50	3–5	0.679	(0.578; 0.798)	<0.001
		60–90	≥6	0.587	(0.526; 0.656)	<0.001
	Number of consultant visits	10–50	0–1	1 (ref.)		
		60–90	≥2	0.642	(0.579; 0.712)	<0.001
d.	Total cost (USD)	10–60	<3491	1 (ref.)		
		70	3491–4765	1.580	(1.391; 1.794)	<0.001
		80	4765–7300	2.185	(1.947; 2.452)	<0.001
		90	≥7300	4.181	(3.809; 4.589)	<0.001
e.	Total cost (USD)	10–60	<3491	1 (ref.)		
		70	3491–4765	1.376	(1.204; 1.573)	<0.001
		80	4765–7300	1.919	(1.697; 2.170)	<0.001
		90	≥7300	3.430	(3.055; 3.851)	<0.001

^a^ Models: (a) each HRU parameter (separately), accounting for repeated measurements for the same patient; (b) all HRU parameters (together), accounting for repeated measurements for the same patient; (c) all HRU parameters (together), accounting for repeated measurements for the same patient and adjusted for confounders: age, renal diseases, obesity, peripheral vascular disease, chronic obstructive pulmonary disease, neurological disorders, malignancy, anemia, schizophrenia/psychosis, gastro-intestinal bleeding, alcohol/drug addiction, left ventricular dysfunction, left ventricular hypertrophy, mitral regurgitation, pulmonary hypertension, healthcare resource utilization one year prior to acute myocardial infarction, type of acute myocardial infarction, compliance to the medical treatment during the first year after acute myocardial infarction, encounter year and years since acute myocardial infarction; (d) costs, accounting for repeated measurements for the same patient and (e) costs, accounting for repeated measurements for the same patient and adjusted for the confounders (see above). ^b^ Number of emergency department (ED) visits not resulted hospitalizations. Abbreviations: CI—Confidence interval, ED—Emergency department, HRU—Healthcare resource utilization, LOS—Length of (hospital) stay, OR—Odds ratio, ref.—Reference group, USD—United States Dollars.

## Data Availability

Data is unavailable due to privacy or ethical restrictions.

## Supplementary Materials

The following supporting information can be downloaded at: https://www.mdpi.com/article/10.3390/jcm12082773/s1, Figure S1: Discriminative ability (ROC curve) of the models for prediction a year of death among post-AMI patients: (a) the model included the variables of all HRU parameters (together), accounting for repeated measurements for the same patient and adjusted for the investigated confounders (see model c Table 3); (b) the model included a total cost, accounting for repeated measurements for the same patient and adjusted for the investigated confounders (see model e Table 3); Table S1: Mean values and deciles of annual healthcare services utilization (HRU) and costs; Table S2: Changes in HRU and cost by approaching to a mortality year (the sub-group analysis included the patients that died during the follow-up period), multivariable models.
