# Supplementary material for: Healthcare Resources Utilization throughout the Last Year of Life after Acute Myocardial Infarction
Source: J Clin Med. 2023 Apr 8;12(8):2773. doi: 10.3390/jcm12082773 (PMC10146999; doi:10.3390/jcm12082773)
Supplement: Supplementary file 1 [file jcm-12-02773-s001.zip › jcm-2270226-supplementary.pdf]

Supplementary Materials

Table S1. Mean values and deciles of annual healthcare services utilization (HRU) and costs.

Parameter	Mean (SD)	Percentiles								
		10	20	30	40	50	60	70	80	90
LOS (Days)	4.08 (14.16)	0	0	0	0	0	2	5	13	14
Number of ED visits[a]	0.332 (2.37)	0	0	0	0	0	0	0	1	2
Number of primary clinic visits	12.98 (11.45)	1	3	6	8	10	13	18	21	28
Number of ambulatory visits	10.39 (21.82)	0	0	1	3	5	6	10	15	25
Number of consultant visits	1.45 (2.23)	0	0	0	0	1	2	2	3	4
Total cost (USD)	4059.60 (9761.90)	160	558	970	1423	1968	2631	3491	4765	7300

[a] Number of emergency department (ED) visits not resulting in admission. Abbreviations: ED – Emergency department, LOS – Length of (hospital) stay, SD – Standard deviation, USD – United States Dollars.

Figure S1. Discriminative ability (ROC curve) of the models for prediction a year of death among post-AMI patients: a) the model included the variables of all HRU parameters (together), accounting for repeated measurements for the same patient and adjusted for the investigated confounders (see model c Table 3); b) the model included a total cost, accounting for repeated measurements for the same patient and adjusted for the investigated confounders (see model e Table 3).

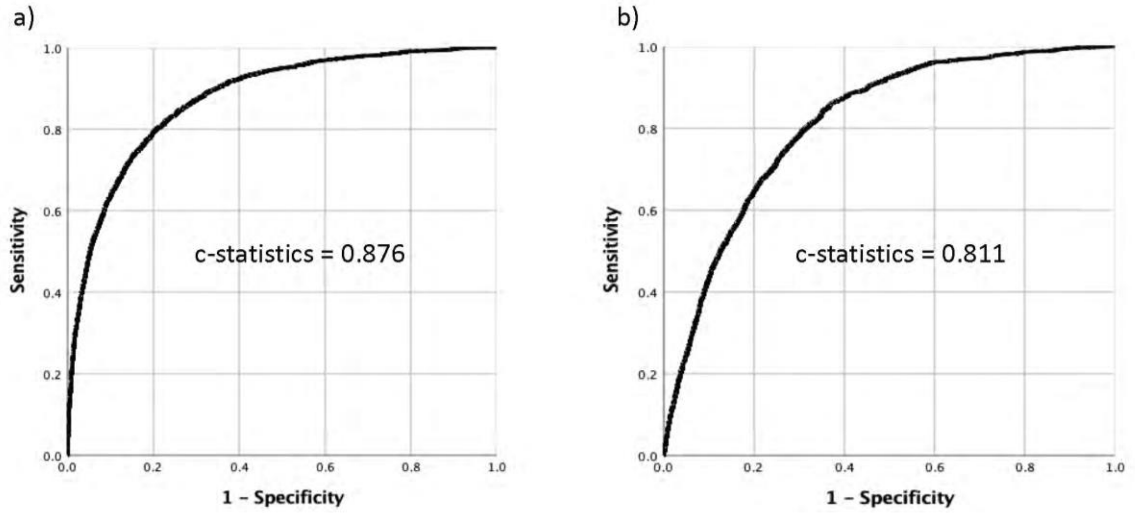

Abbreviations: AMI – Acute myocardial infarction, ROC - Receiver operating characteristic (curve).

Table S2. Changes in HRU and cost by approaching to a mortality year (the sub-group analysis included the patients that died during the follow-up period), multivariable models.

Dependent variable	Model[a]	B (SE)	(95% CI)	p
LOS (Days)	a.	1.062 (0.065)	(0.934 ; 1.190)	<0.001
	b.	1.108 (0.066)	(0.978 ; 1.238)	<0.001
Number of ED visits[b]	a.	0.058 (0.013)	(0.032 ; 0.084)	<0.001
	b.	0.052 (0.019)	(0.014 ; 0.090)	0.007
Number of primary clinic visits	a.	-0.081 (0.054)	(-0.186 ; 0.024)	0.130
	b.	-0.091 (0.050)	(-0.189 ; 0.007)	0.068
Number of ambulatory visits	a.	0.356 (0.111)	(0.138 ; 0.573)	0.001
	b.	0.176 (0.111)	(-0.041 ; 0.393)	0.111
Number of consultant visits	a.	-0.066 (0.009)	(-0.083 ; -0.048)	<0.001
	b.	-0.063 (0.009)	(-0.080 ; -0.045)	<0.001
Total cost (USD)	a.	450.532 (45.832)	(360.702 ; 540.361)	<0.001
	b.	399.929 (53.757)	(294.566 ; 505.291)	<0.001

[a] Models: a) accounting for repeated measurements in the same patient, b) accounting for repeated measurements for the same patient and adjusted also for confounders: age, renal diseases, obesity, peripheral vascular disease, chronic obstructive pulmonary disease, neurological disorders, malignancy, anemia, schizophrenia/psychosis, gastro-intestinal bleeding, alcohol/drug addiction, left ventricular dysfunction, left ventricular hypertrophy, mitral regurgitation, pulmonary hypertension, healthcare resource utilization one year prior to acute myocardial infarction, type of acute myocardial infarction, compliance to the medical treatment during the first year after acute myocardial infarction, encounter year and years since acute myocardial infarction. [b] Number of emergency department (ED) visits not resulted hospitalizations. Abbreviations: B – Regression coefficient (B), CI – Confidence interval, ED – Emergency department, HRU - Healthcare resource utilization, LOS – Length of (hospital) stay, OR – Odds ratio, ref. – Reference group, SE – Standard error, USD – United States Dollars.
